# Methodologic Issues and Approaches to Spatial Epidemiology

**DOI:** 10.1289/ehp.10816

**Published:** 2008-04-25

**Authors:** Linda Beale, Juan Jose Abellan, Susan Hodgson, Lars Jarup

**Affiliations:** 1 Small Area Health Statistics Unit, Department of Epidemiology and Public Health, Imperial College London, London, United Kingdom; 2 CIBER Epidemiología y Salud Pública (CIBERESP), Spain; 3 Institute of Health and Society, Newcastle University, Newcastle Upon Tyne, United Kingdom

**Keywords:** disease mapping, environmental epidemiology, geographic information systems (GIS), risk analysis, spatial epidemiology, uncertainty

## Abstract

Spatial epidemiology is increasingly being used to assess health risks associated with environmental hazards. Risk patterns tend to have both a temporal and a spatial component; thus, spatial epidemiology must combine methods from epidemiology, statistics, and geographic information science. Recent statistical advances in spatial epidemiology include the use of smoothing in risk maps to create an interpretable risk surface, the extension of spatial models to incorporate the time dimension, and the combination of individual- and area-level information. Advances in geographic information systems and the growing availability of modeling packages have led to an improvement in exposure assessment. Techniques drawn from geographic information science are being developed to enable the visualization of uncertainty and ensure more meaningful inferences are made from data. When public health concerns related to the environment arise, it is essential to address such anxieties appropriately and in a timely manner. Tools designed to facilitate the investigation process are being developed, although the availability of complete and clean health data, and appropriate exposure data often remain limiting factors.

In this article, we review some of the major limitations facing spatial epidemiology and discuss emerging techniques that can help overcome some of these issues. We introduce readers to several tools recently developed to enable epidemiologists and public health practitioners to undertake increasingly sophisticated spatial analyses. Such tools form one part of an increasing number of national environment and public health tracking programs, which are being set up to encourage and exploit the linkage of environmental and health data.

The assessment of risk of adverse health effects from environmental hazards and the analysis of the geographic variation of disease risks as well as cluster detection are areas of increasing public interest and draw on research from a number of different disciplines (Elliott et al. 2000; Rushton and Elliott 2003). Such disease-mapping, cluster, and risk assessment studies require both accurate and detailed population and health data, as well as clearly defined exposure assessments (Jarup 2004; Nieuwenhuijsen et al. 2005). The increased availability of spatial environmental, health, and population data combined with improved statistical methods and spatial analysis techniques has fueled an increase in spatial epidemiologic studies, which assess the geographic distribution of potential health risks and their association with environmental risk factors. The ability to rapidly locate disease clusters, assess the spatial distribution of disease risk, and link environmental data and health outcomes provides a powerful tool for the evaluation of spatial relationships between disease and environmental hazards.

The availability and quality of geographically referenced data on population, health outcomes, and environmental risk factors are crucial to any spatial epidemiologic analysis. Preferably, those data will have exact and accurate associated spatial and temporal information, but very often such data are available only as aggregated summaries. Ideally, detailed information for a study population would be used, including individual characteristics, movements, personal exposures, and subsequent health records. Although individual-level health data may exist, confounder information and exposure data rarely, if ever, exist at the individual level, so some simplifications must be imposed (Elliott et al. 2000). Local geographically linked health and population data are, however, becoming increasingly available, which enables the analysis of small-area variations in health risk. Such data offer some advantages and challenges to spatial risk assessment.

## Developing Statistical Approaches to Spatial Epidemiology

Because of data limitations, most spatial epidemiology studies use data aggregated at the area level. Several statistical techniques and tools are available to calculate area-level risks together with confidence intervals as a measure of the uncertainty associated with the estimated risks. Standardizing summary rates allows the effect of known risk factors, such as age, sex, and socioeconomic status, to be taken into account and can take the form of indirect or direct standardization.

The most common summary measure for mapping disease risk is the standardized morbidity/mortality ratio (SMR). This method of indirect standardization, which compares the number of cases observed in the study population with the number of cases expected using age-specific rates from a standard population, can be problematic in small areas or in cases of rare diseases. In these situations, the estimates will be dominated by sampling variability (Elliott et al. 2000). The dependence of the age and sex weights in the structure of the study population (denominator value) means that SMR measures are not directly comparable between different exposure groups and should not, therefore, be used in cases where the population structure is significantly different between the comparison groups.

An alternative would be to use so-called comparative mortality figures, which compare the number of expected cases in the standard population (derived from strata-specific disease rates in each exposure group) with the number observed. Such direct standardization allows valid comparison of risks in different exposure groups, but does require substantial numbers of cases in each exposure category and accurate assessment of the age-specific incidence proportions for the study population observed. For rare diseases or at the small geographic level, numbers of cases are usually so few that the incidence proportions observed may be statistically unstable (Jarup and Best 2003), and SMRs are usually used, although the caveats noted above should be borne in mind.

Confidence intervals are usually reported together with the risk measure provided. The calculation of confidence intervals relies on either the statistical assumption of approximate normality for the distribution of the risk indicator, or a suitable mathematical transformation (e.g., logarithmic). While an assumption of approximate normality may hold when working with large numbers, this will not be true when sparseness exists. When using low count data, it is common to use an “exact approach” to calculate confidence intervals, which is based on the Poisson distribution (Esteve et al. 1994).

The investigation of the spatial variation in disease outcome patterns is also important in locating the areas with highest risk in epidemiologic analysis. This is usually done using either “global” or “focalized” clustering analyses, or disease mapping. In the context of epidemiology, clustering considers the spatial aggregation of disease cases in relation to the pattern of noncases or population at risk. A disease is said to show spatial clustering if there is any residual spatial variation in risk beyond that explained by the background population reference. This residual variation, also known as overdispersion, may be attributable to either a true extra-aggregation of the cases in one or more areas, or to dependence between the observations. Overdispersion can be caused by a number of factors, such as an environmental hazard, the existence of an infectious agent, or variations in genetic susceptibility, all of which may vary spatially. The presence of overdispersion is usually explored using hypothesis tests that check for homogeneity (e.g., chi-square test), autocorrelation [e.g., test based on Moran’s index (Moran 1950)], or both [e.g., Tango’s test (Tango 1995, 2000)]. Cluster location techniques are also based on hypothesis-testing methods, whereby the study region is literally scanned for clusters by superimposing a number of circular (or elliptical) windows to determine the group of contiguous areas with the most significant excess risk (Besag and Newell 1991; Kulldorff and Nagarwalla 1995; Openshaw et al. 1987). Individual data (e.g., case–control data) can also be used to analyze the spatial variation of disease risk. Indeed, versions exist of both Besag and Newell’s (1991) and Kulldorff and Nagarwalla’s (1995) methods for dealing with individual data in cluster location studies. The estimation of disease risk surfaces, using statistical point process techniques with case–control data (Kelsall and Diggle 1998), offers another approach. In this method, the surface of risk is obtained as the ratio of the intensity functions of both patterns (estimated using kernel-based methods), and the level of clustering in both cases and controls is compared using Ripley’s K function (Ripley 1981).

Disease-mapping methods deal with the estimation of the spatial distribution of disease risk. Small-area analyses can also mean that data are sparse; small populations often have small numbers of observed and expected health outcomes and, therefore, unstable risk estimates. Bayesian hierarchical models have significantly helped to cope with sparseness in disease-mapping studies (Best et al. 2005). These models shrink unstable risks toward the local mean risk by “borrowing” information between areas. This results in an adaptive smoothing approach whereby risks in areas with more information (e.g., urban areas) are less smoothed than in areas that exhibit higher sampling variation (typically those with low number of cases), and thus produce more stable estimates of the pattern of underlying disease risk (Richardson et al. 2004). However, although raw risks can produce “noisy” maps that are difficult to interpret, oversmoothed maps may produce a homogeneous risk surface, masking the true risk distribution.

Ecologic regression models are commonly used to assess association between risk factors and health outcomes at the area level. The overdispersion phenomenon mentioned above prevents the use of standard generalized linear models (GLM) (McCullagh and Nelder 1989) such as Poisson and logistic regression that assume independence between observations. Ignoring spatial autocorrelation, often present in this type of data, may lead to biased estimation of the regression coefficients and underestimation of the uncertainty surrounding them (i.e., falsely) narrower confidence intervals (Schabenberge and Gotway 2005). To address this problem, GLMs are usually extended by including random effects in the linear predictor. These random effects are in turn assigned a joint normal multivariate distribution whose covariance matrix models the spatial autocorrelation structure. These models are a particular class of multilevel or hierarchical models and inference can be made under the Bayesian or frequentist paradigm (Schabenberge and Gotway 2005). In the latter case, depending on whether the inference is made conditionally or marginally, this extension leads, respectively, to generalized linear mixed models (McCulloch and Searle 2001) or to generalized estimated equations (Hanley et al. 2003).

## Developing Geographic Approaches to Spatial Epidemiology

The spatial component of health data can play a crucial part in helping explain variability in risk because health status, environmental hazards, population numbers, demographic and socioeconomic profiles, and other relevant characteristics (e.g., susceptibility and exposures) all vary across space. In this sense, geographic space varies uniquely in different locations and at different times and creates unique places in which people live and work. Geography defines the spatial context and character in which health risks occur. Any movement between places is significant (Wakefield et al. 2001); pollution and other hazards that form concentrations can be modified as they move through the environment and affect different places in different ways, whereas spatial patterns in risk will be complicated by differences in susceptibility and by variations that arise simply because populations are unique to particular places.

The importance of geographic information science is increasingly recognized in relation to spatial epidemiologic research because it provides the fundamental geographic context to exploring spatial patterns in data. A geographic information system (GIS) provides an integrated set of tools that allow both the analytical manipulation and the visual representation of spatial data. In the context of epidemiology and public health, this provides a powerful aid to the analysis and understanding of the relationships between geography, the environment, and human health. Geographic information science and GIS are also being increasingly relied upon for exposure assessment. GIS can be used for simple spatial analysis, in line with Tobler’s first law of geography (Tobler 1970), which states that all things are related, but near things are more strongly related than distant ones; or can be extended to data analysis algorithms that allow spatial analysis with more complex models. Linking exposure and disease began with simple location mapping, and to date, most epidemiologic analyses have used only simple spatial interrogation and analysis, for example, using distance measurements to identify at-risk populations from known point pollution sources such as industrial plants and landfill sites (Aylin et al. 2001; Elliott et al. 2001; Hodgson et al. 2004), linear sources such as roads and rivers (English et al. 1999; Verkasalo et al. 2004), and modeled pollutant dispersion (Hodgson et al. 2007; Ihrig et al. 1988). More complex data analysis algorithms for estimation, prediction, and simulation have been proposed (Openshaw 1998) and are increasingly being implemented, often as additional tools developed for use within proprietary GIS software. Despite developments in this area and the increasing recognition of spatially explicit processes in determining disease risk, the use of spatial information beyond recording spatial location and mapping disease risk remains rare.

## Developing Tools for Spatial Epidemiology

Increasingly, software tools that integrate specialist statistical methodologies and spatial analysis are being developed for use in epidemiology and public health. Many of the advanced statistical methods of cluster investigation and disease mapping are not part of the routine knowledge of the public health specialist (Morris and Wakefield 2000; Waller and Lawson 1995), and the ability to access tools that automate such complex techniques can assist in the advancement of epidemiologic analysis. The “black box” nature of such models might be seen in a negative light, and can potentially lead users to be less aware of their data or how they are being analyzed, but in many cases these models offer excellent data exploration tools. It should be noted, however, that these tools cannot overcome the numerous issues inherent with small-area spatial epidemiology, nor can meaningful output be produced with poor-quality exposure, health, or population data. Nonetheless, in many cases they can offer a significant time advantage over traditional methods of analysis.

In this section we outline several tools that have been developed for epidemiology or public health. This list concentrates on tools that allow, as a minimum, some mapping function, but specifically some spatial analysis of data. This list is by no means exhaustive, and a number of other spatial statistical software packages are widely used in spatial epidemiology and public health that allow connection to GIS or that produce results that can be subsequently displayed in a mapping package or GIS [e.g., SaTScan (http://www.satscan.org), Clusterseer 2 (http://www.terraseer.com/products_clusterseer.php), Epimap (http://www.cdc.gov/epiinfo/about.htm)].

HealthMapper was developed by the World Health Organization as a tool for surveillance and disease mapping (World Health Organization 1999). Its main focus is on African countries, where it is being used in programs to control infectious diseases, including Guinea worm disease, malaria, HIV/AIDS, leprosy, and tuberculosis. It consists of three components: a standardized geographic database with information on boundaries at different geographic levels (e.g., community, village) as well as on demography; a data manager, which is an interface between the core geo-referenced database and user-supplied databases that also allows the creation of reports and summary tables; and a mapping interface with interactive maps and graphs for visualization of information. The system’s capabilities for epidemiologic analysis are rather limited because it is more oriented to the descriptive component of the distribution and magnitude of health risks and their determinants. It runs on a Windows operating system, and distribution is either free or inexpensive based on institutional agreements with the World Health Organization.

SIGEpi [Sistemas de Información Geográfica en Salud (Martinez et al. 2001)] was developed by the Pan-American Health Organization to strengthen the analytical resources in epidemiology and public health in the region of the Americas. It includes a number of tools that allow interrogation of spatial data, as well as methods for analyzing data on health outcomes and determinants, such as descriptive and exploratory techniques, smoothing models for disease mapping, spatial clustering, and construction of composite health indexes. Risk analysis can be carried out to assess the association between environmental indicators and health outcomes, at both individual and aggregated levels. It also has an interface to identify critical areas or population subgroups using complex conditional expressions based on covariates and indicators, and geographic analysis tools. The software is distributed with an inexpensive license and runs on a Windows operating system.

GeoDa, developed at the Spatial Analysis Lab, University of Illinois (https://www.geoda.uiuc.edu/), was originally intended to provide a link between statistical software and Environmental Systems Research Institute’s ESRI ArcView (version 3.x; ESRI, Redlands, CA, USA) GIS, but it has since been developed as a standalone application, written in C++, that works under any Microsoft Windows–compliant operating system (Anselin 2003). It offers a number of spatial analysis functions and mapping tools, including the calculation of raw rates (as a ratio of event count to base population at risk) and relative risk or excess risk (as a ratio of observed events over expected). Furthermore, the rates can be smoothed using three different methods: empirical Bayes, a spatial window average (using total number of events in the window), and spatial empirical Bayes using the window average as the reference of adjustment (rather than the overall mean).

**Figure 1:**
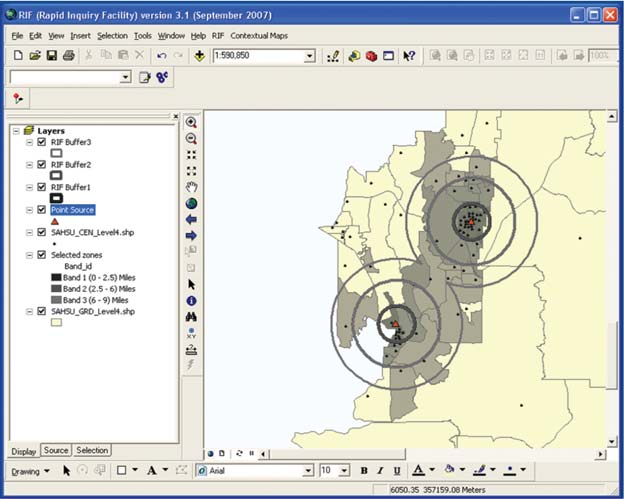
Risk analysis using the RIF.

The Rapid Inquiry Facility (RIF) developed at the U.K. Small Area Health Statistics Unit integrates advanced methods in statistics, spatial analysis, and spatial epidemiology to allow assessment of the health risks related to environmental exposure, producing disease maps with and without statistical smoothing. The RIF was originally intended to facilitate the estimation of risks for any given condition for a population within defined areas around a point source, relative to the local population in a local reference region within the United Kingdom (Aylin et al. 1999). The RIF was further developed for use in Europe in the European Health and Environment Information System for Exposure and Disease Mapping and Risk Assessment (EUROHEIS) project (EUROHEIS 2003). Within the framework of the U.S. Centers for Disease Control and Prevention’s Environmental Public Health Tracking (EPHT) program (http://www.cdc.gov/nceh/tracking/), the RIF has been redeveloped in Visual Basic and works as an application that is embedded in ESRI ArcGIS (version 9 and higher). The RIF takes advantage of open database connectivity to connect to an external database where geocoded health and population data are stored. These data sets are then used for analysis, and results are displayed in the GIS. The risk analysis allows the user to calculate rates and relative risks within user-defined distance bands or other user-defined areas around one or more point or area sources (Figure 1). The disease-mapping functionality of the RIF allows a user to produce maps of directly standardized rates and indirectly standardized risks. It also allows smoothing of the relative risks via empirical Bayesian estimation. The RIF is currently being further developed to include visualization of spatial uncertainty and integration with other relevant software, such as WinBUGS and SaTScan. It runs on a Windows operating system, and it is intended that the software will eventually be distributed as freeware.

## Methodologic Issues and Advances

### Data problems

As stated above, data limitations often require that spatial epidemiology analyses be carried out at an ecologic level. Although ecologic studies can be useful for detecting associations between exposure distributions and disease occurrence, the use of aggregated data does have associated problems.

Results can be affected by selection bias if the underlying population and health data are inaccurate and incomplete. Although census data may be available at a detailed level of (dis)aggregation (e.g., output area, census block group, municipalities, etc.), in many countries census areas are large, population data are not released, and censuses are unreliable. In addition, such data are usually collected for a single snapshot in time, every decade, for instance, meaning any changes in populations between census counts will add to the uncertainty and unreliability in these data. An important source of this type of bias is the ascertainment of health registries, which often exhibit geographic as well as temporal variation (Forand et al. 2002). With rare health events, these errors or variations in the small-area health and population counts can result in major uncertainties.

Spurious associations may also be due to information bias because generally the data used in spatial epidemiology studies, and specifically exposure data, are actually proxies that will not have been collected for that purpose and therefore may well not offer the most appropriate information. Misclassification can also occur because of inaccuracies in the location of cases and populations, which would directly affect the validity of any epidemiologic study, potentially introducing spurious temporal or spatial patterns in risk (Bonner et al. 2003; Oliver et al. 2005). The effect of any geocoding errors will depend on the spatial variation of the population or sources of risk, although greater positional errors are likely in rural rather than urban addresses (Ward et al. 2005). Population migration will introduce exposure misclassification and potentially introduce errors in temporal or spatial patterns in risk (Arnold 1999). This is especially problematic for outcomes with long latency periods between exposure commencement and disease onset, but in many cases, the latency periods, migration, and relevant exposure metrics are not well characterized. In exposure terms, both differential and nondifferential misclassification can occur, for example, when environmental measures do not accurately reflect actual exposure, and may lead to biased study results and/or a reduction in study power.

Because boundary data used for population and health data in epidemiologic studies tend to be administrative boundaries rather than physical boundaries, the boundary locations can, and do, change over time. Area names and codes can also change, which can be further complicated by the fact that different government departments can develop different coding systems for administrative geographies, or use slightly different names for the same area. Inconsistent geography is problematic for any spatial and/or temporal study that spans time periods when boundary changes have occurred and is a major problem when trying to produce and compare meaningful statistics over time.

The geographic resolution at which the study is carried out may also have an impact on the results. Health risks are often mapped to relatively arbitrary administrative areas (e.g., the level at which population and covariate data are available), but risks can be sensitive to changes in the scale of output, known as the “modifiable area unit problem” (Openshaw 1984). Grouping data at different levels of spatial resolution (e.g., wards, census tracts, regions) or aggregating data to different areal arrangements will inevitably lead to variation in the results, which may affect the interpretation of the findings.

In the context of cluster analysis, the impact of boundary tightening (also known as the Texas sharpshooter effect) must be considered. A narrowly defined underlying population will give rise to a lower number of expected cases and a greater estimated excess risk. The effects of boundary tightening will be associated with the selection of the study area, time frame, age and sex groups, and diagnostic categories. When investigating risks around a putative source of pollution, although there might be a basis for investigating the population living in very close proximity to the source, thought should be given to whether the size of this “exposed” population is sufficient to provide a meaningful risk estimate. Although a power calculation might not be appropriate in a cluster analysis, some consideration as to the likely statistical significance of the observed effect should be given to establish how informative the results are likely to be. Detecting whether an identified “cluster” has any epidemiologic significance and even identifying causation are rarely possible post hoc. Ideally, cluster investigations should be confined to highly specific exposure–disease associations with high anticipated relative risks (Olsen et al. 1996).

Results obtained solely from aggregate (ecologi) data should not be used for making assumptions about the nature of an association at the individual level (ecologic fallacy). Factors associated with national or regional disease rates may not necessarily be associated with disease in individuals (Morgenstern 1998). Using small-area data reduces some of the components of ecologic bias created by within-area heterogeneity but by no means rules this bias out. Small-area studies also allow local effects (e.g., impacts of point sources of pollution) to be investigated (Elliott and Wartenberg 2004). In terms of exposure data, one of the strengths of the group-level data is that they can be more accurate than the corresponding individual exposures (Richardson 1992). Indeed, for certain exposure measures, misclassification in the group estimate will have less of an influence on the resultant risk estimates than will misclassification in the individual estimate (Armstrong 2004).

Advances in statistical methods in the last decade include the extension of spatial disease-mapping models to incorporate the time dimension (see, e.g., Knorr-Held 2000; Waller et al. 1997). They aim at splitting the relative risk into main spatial and temporal effects as well as space–time interaction. In the same spirit of the purely spatial models, “strength” of information is borrowed across time points, as well. The use of space–time models to investigate patterns of disease is discussed in detail by Abellan et al. (2008). More recent is the joint analysis of two or more related diseases in space (Held et al. 2005; Knorr-Held and Best 2001) or in space and time (Richardson et al. 2006), where again the borrowing of information is allowed across diseases, in addition to space and time. Ecologic studies have also benefited from the combination of individual- and area-level information recently proposed by Wakefield (2004) and Jackson et al. (2006). This new multilevel methodology aims to obtain the individual-level effects of exposure in disease risk using the area-level data supplemented with small samples of individual-level data. Although an analysis based on area-level data may be subject to ecologic bias, an analysis based on individual-level data would lack the statistical power needed to analyze the small-area variation on disease risk. The combination of both corrects the ecologic bias in the estimated effect while preserving the statistical power of the analysis.

### Uncertainty

Interpretation and decision making with spatial data should be done with knowledge of their nature and quality or reliability (Buttenfield and Beard 1994; Longley et al. 2005). From the acquisition of data from recorded physical features through to geo-visualization, information may undergo a number of transformations to produce derived data. Data may be converted between feature type (e.g., point, line, or area), interpolated, simplified, sampled, or quantified, particularly because spatial representation is usually a decision of the analyst rather an inherent feature of the data. Derived data can then be displayed using a variety of geo-visualization techniques (Cheung and Shi 2004; Schneider 2001). Any of these stages may affect uncertainty associated with the data.

**Figure 2:**
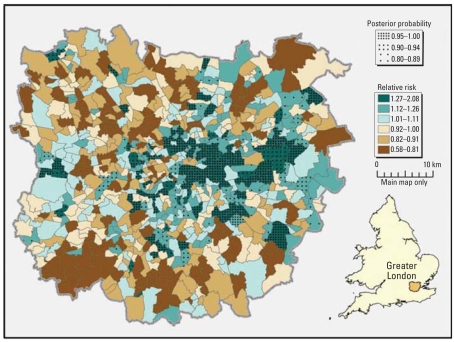
Smoothed risk of lung cancer incidence, with posterior probabilities: Greater London, ward level, 1999–2003.

It is common practice in epidemiology to present relative risks with confidence intervals, but results of spatial epidemiologic analyses are often presented as mapped relative risks with no attempt to report the uncertainty in the risk estimates. The Bayesian approaches mentioned above provide a rich output in terms of uncertainty measures associated to the relative risks, such as 95% credibility intervals and ranks of the risks (Ferrándiz ks being > 1 (Jarup et al. 2002). The latter were used by Richardson et al. (2004) to propose rules for detecting areas with high risk; the authors proved that they are highly specific while having reasonable sensitivity. Whichever measure is preferred, uncertainty should be reported to help the interpretation of the relative risks. Figure 2 shows an example of output from the RIF that combines the posterior abilities with the smoothed relative risks.

Where information exists to quantify uncertainty, a number of different techniques can be used to incorporate these data with other spatial data to facilitate interpretation. A single bivariate choropleth map can convey information about geographic variation of risk estimates combined with their accompanying uncertainty (Monmonier 2006). Effective use of color is crucial to producing readable bivariate maps; for example, opacity can be used to clearly represent uncertainty (Drecki 2002). Bivariate choropleth maps will be less effective in cases where a large number of different classes are required. Several conceptually simple methods can also be employed to effectively combine uncertainty with spatial estimates, including color saturation, where highest uncertainty is depicted by desaturated colors (de Cola 2002), or decreasing boundary crispness with increased uncertainty. Multiple maps can be used to report uncertainty separately either in a static form or including animation of the spatial distribution of risk values (Goovaerts 2006).

Following the work of Richardson et al. (2004), posterior probability values can be used to aid interpretation of areas of actual excess risk. Areas where the relative risk is > 1 and the posterior probabilities are > 0.8 can be more confidently considered as having high risk.

In some cases, there may be a need to introduce uncertainty cartographically. Introducing some positional error or aggregating data can be used to preserve spatial anonymity. These data could then be used to produce detailed disease-distribution maps, but the needs of epidemiology must be very carefully balanced with demands to preserve individual privacy (Leitner and Curtis 2004), particularly in the case of dot mapping, where a point is assumed to depict an exact location.

## Conclusions

The increase in data availability, methods, and technology is clearly important for the future of spatial epidemiology, but it also presents significant challenges. Using approaches from a number of different disciplines, such as statistics and geographic information science, epidemiologic studies demand a diverse approach and consequently higher demands on personnel. Multidisciplinary teams must work closely to ensure that adopted approaches are fully appreciated and complement each other, rather than merely introducing or, at worst, multiplying errors. For institutions and health departments handling public health concerns related to the environment, this carries an appreciable cost.

The availability of software tools designed to facilitate the investigation process is key to efficiently handling issues where public health concerns related to the environment arise. The ability to use specialist statistical methodologies and GIS without requiring detailed knowledge of such approaches enables complex analysis to be carried out without unreasonable demands in terms of expertise and time. Consequently, understanding the local data issues and the interpretation of the analytic output can remain a crucial component of the task, rather than being diluted by the overwhelming demands on time and cost of undertaking the analyses. These software tools cannot overcome the numerous data issues detailed in this article but can go some way toward more rapidly linking and analyzing environmental and health data.

The availability of geocoded data provides great opportunities for epidemiologic research, offering the ability to carry out largescale studies over long time periods. Data are rarely collected specifically for epidemiologic research and therefore may not be completely appropriate for the analysis being undertaken. Analysis must consider not only the appropriateness of the method and the available data, but also the inevitable simplification that occurs when attempting to model real-world phenomena.

Initiatives to build nationwide tracking programs that provide integrated health and environmental data have recently begun. In the United States, the Centers for Disease Control and Prevention’s EPHT program identified that there were no existing systems, at either the state or the national level, that enabled linkage and, therefore, monitoring of relationships among hazards, exposures, and health effects. This initiative recognizes the importance and future need of a more standardized approach to data collection and storage. In the United Kingdom, the Health Protection Agency has also proposed to create a national environmental health tracking system that links environmental chemicals, health, exposure, and other factors in an effort to better understand the burden of disease attributable to environmental factors in the United Kingdom. The scope and importance of such schemes should not be underestimated because they not only provide sources for suitable data and tools for epidemiology but also lead to a more specific, integrated, and standard approach to data collection and analysis. Effective epidemiologic analysis of data trends over time and space can help drive public health policy.
